# Supplementary material for: Cooperating yet distinct brain networks engaged during naturalistic paradigms: A meta-analysis of functional MRI results
Source: Netw Neurosci. 2018 Oct 1;3(1):27–48. doi: 10.1162/netn_a_00050 (PMC6326731; doi:10.1162/netn_a_00050)
Supplement: Supplementary file 1 [file netn-03-27-s001.pdf]

Bottenhorn, K. L., Flannery, J. S., Boeving, E. R., Riedel, M. C., Eickhoff, S. B., Sutherland, M. T., & Laird, A. R. (2018). Supporting Information for "Cooperating yet distinct brain networks engaged during naturalistic paradigms: A meta-analysis of functional MRI results." *Network Neuroscience, 3*(1), 27–48. https://doi.org/10.1162/netn_a_00050

SUPPLEMENTAL MATERIAL

Supplementary Table 1. PubMed IDs included in the final analysis

9831460	17395609	20521850	22609273
11467917	17412611	20570684	22745788
11798274	17504783	20576388	22759716
11870922	17631871	20738888	22952277
11923436	18412134	20804287	23202431
12419129	18608980	20949046	23209583
12628177	18768725	21111828	23238964
12644355	18801445	21148091	23414211
12814585	18809234	21248113	23558102
15006683	18823249	21305666	23616340
15038005	18952070	21382559	23734202
15091347	19015097	21484518	23978654
15165355	19244522	21596764	24015241
15291723	19279244	21612817	24080372
15511655	19300986	21749711	24194828
15800188	19376237	21749924	24333752
15809000	19500000	21818311	24583253
15890534	19531876	21861684	24814646
15893474	19585588	21972849	24845161
15935262	19596123	22019857	24949579
16167193	19733672	22038718	24972303
16338048	19780040	22110619	25460498
16436599	19781579	22116038	25662868
16467858	20039023	22248574	25716010
16604320	20121935	22432905	25762672
16628606	20132831	22504276	18240326
16687157	20146607	22516367	
17298595	20378581	22522473	

These represent the papers from which data were extracted for the described analyses. From these 110 papers, we mined information about the experimental design and resultant statistical analyses to assess the neural substrates of naturalistic behavior.

Supplementary Table 2. Coordinates and region labels for each MAG's cluster centroids

Cluster #	Voxels (mm^3)	Center of Mass (MNI Coordinates)			Mean Z-score	Region		BA
		x	y	z				
MAG 1								
1	14104	54	-58	7	5.14	R	Middle temporal gyrus	
2	10743	-50	-63	10	5.20	L	Middle temporal gyrus	
3	1077	-20	-85	38	3.92	L	Cuneus	
4	921	48	6	45	3.85	R	Middle frontal gyrus	6
5	856	25	2	58	4.02	R	Superior frontal gyrus	6
6	768	7	-51	57	3.79	R	Precuneus	
7	512	-10	-91	6	3.77	L	Lingual gyrus	17
MAG 2								
1	3884	-56	-48	6	4.25	L	Middle temporal gyrus	
2	2704	-48	20	20	4.19	L	Inferior frontal gyrus	9
3	2472	-2	11	63	4.11	L	Superior frontal gyrus	6
4	2237	-10	57	30	4.61	L	Superior frontal gyrus	
5	2012	-54	4	-22	4.41	L	Middle temporal gyrus	
6	1882	-3	-56	35	4.08	L	Precuneus	
7	1523	-46	31	-7	4.37	L	Inferior frontal gyrus	
8	1216	54	-10	-20	3.77	R	Superior temporal sulcus	
9	983	-47	2	49	4.05	L	Precentral gyrus	6
10	839	53	24	20	3.58	R	Middle frontal gyrus	46
11	725	59	-57	14	4.42	R	Middle temporal gyrus	
12	641	-56	-24	-14	4.18	L	Middle temporal gyrus	
MAG 3								
1	8277	-17	-8	-9	4.41	L	Amygdala	
2	6121	23	-4	-15	4.77	R	Amygdala	
3	1135	-46	22	-3	3.82	L	Inferior frontal gyrus	
4	860	-53	-64	-6	4.08	L	Middle occipital gyrus	
5	853	1	-29	-15	3.85	L	Periaqueductal gray	
6	765	44	-55	-21	3.96	R	Cerebellum (culmen)	
7	744	-42	-57	-19	3.56	L	Fusiform gyrus	37
8	521	1	20	30	3.52	L	Cingulate gyrus	
9	461	-32	9	-23	3.47	L	Inferior frontal gyrus	
10	5	-8	-9	-4	3.65	L	Hypothalamus	
MAG 4								
1	8391	-16	-56	9	4.32	L	Posterior cingulate gyrus	
2	6688	21	-49	3	4.50	R	Parahippocampal gyrus	30
3	1782	9	-88	13	3.97	R	Cuneus	18
4	1411	40	-70	38	4.05	R	Angular gyrus	
5	810	-30	55	-3	4.18	L	Middle frontal gyrus	10
6	740	27	59	5	3.87	R	Middle frontal gyrus	
7	21	17	-71	34	3.36	R	Cuneus	
MAG 5								

1	15817	56	-12	2	5.11	R	Superior temporal gyrus	
2	12334	-55	-19	5	5.71	L	Superior temporal gyrus	41
MAG 6								
1	8781	-23	-61	50	4.08	L	Superior parietal lobule	
2	7597	22	-65	52	4.63	R	Precuneus	7
3	5153	-23	-87	6	3.96	L	Middle occipital gyrus	7
4	4501	-30	-8	56	4.50	L	Precentral gyrus	
5	2846	27	-1	57	4.59	R	Superior frontal sulcus	
6	2481	34	-80	21	3.95	R	Middle occipital gyrus	7
7	1430	43	-68	-5	3.66	R	Lateral occipital sulcus	18
8	1199	15	-89	-4	3.66	R	Lingual gyrus	
9	1176	46	9	33	4.12	R	Precentral gyrus	
10	328	-20	-28	-6	3.74	L	Parahippocampal gyrus	6
11	151	-17	-25	8	3.29	L	Thalamus	
12	5	20	-80	-18	3.18	R	Cerebellum (declive)	
13	1	26	12	52	3.09	R	Superior frontal sulcus	

Labels of the center-of-mass coordinates per cluster for each MAG. Clusters are not restricted to each centroid's label, but may extend into neighboring gyri and sulci. For a more thorough understanding of the extent of each cluster, refer to *Meta-Analytic Groupings* in the Results and see Figure 2.

Supplementary Table 3. Modality distribution across MAGs

Modality	MAG 1	MAG 2	MAG 3	MAG 4	MAG 5	MAG 6	MAG 7	Total
Audiovisual	28	10	26	16	18	34	21	153
Auditory	4	12	4	1	5	16	9	51
Pain	0	2	3	2	2	0	0	9
Tactile	1	2	2	0	0	0	4	9
Visual	23	19	7	22	32	4	43	150
Total	56	45	42	41	57	54	77	372

The list of stimulus modalities that were present in the corpus, as well as the distribution of experiments based on stimuli using each sensory modality across MAGs. Total count per term and per MAG are included to provide a scale for comparison across modalities and MAGs.

Supplementary Table 4. Stimulus types across MAGs

Modality	MAG 1	MAG 2	MAG 3	MAG 4	MAG 5	MAG 6	MAG 7	Total
Film	35	13	19	21	18	32	31	169
Virtual Reality	15	12	14	18	30	4	27	120
Speech	2	11	2	1	4	7	5	32
Music	1	4	2	0	0	11	3	21
Video Game	0	3	3	1	2	0	4	13
3D image	1	0	0	0	1	0	4	6
Tactile	1	2	2	0	0	0	1	6
Picture	0	0	0	0	2	0	2	4
Sounds*	1	0	0	0	0	0	0	1
Total	56	45	42	41	57	54	77	372

Stimulus types that were used across the naturalistic corpus and how they were clustered into MAGs based on the results of the clustering of corresponding activation patterns from each experiment in the corpus. *Sounds was excluded from Figure 2, as its low total term count caused a lack of readability in the graphical presentation and the stimulus category itself contributed very little to the overall discussion of the MAGs.

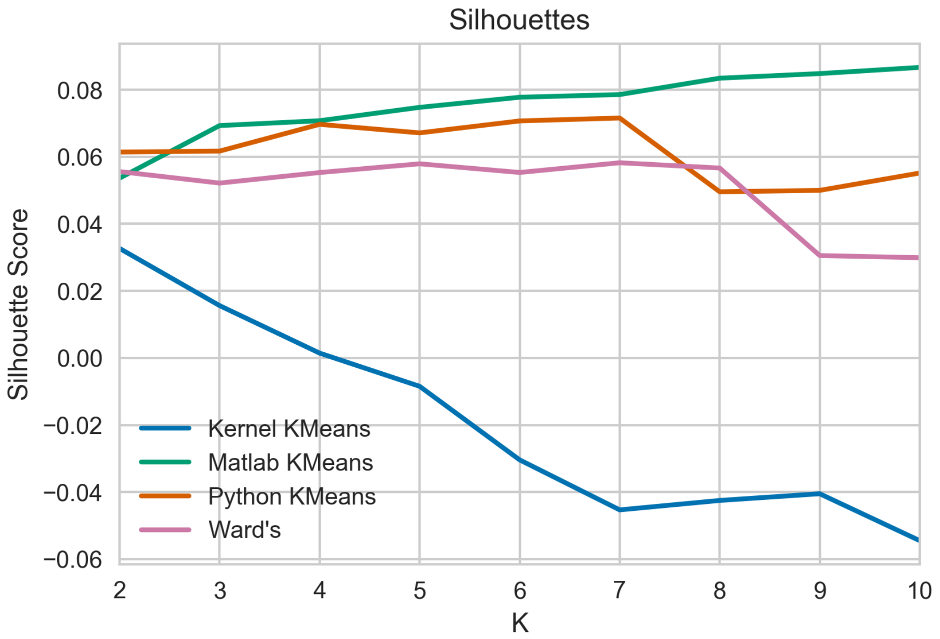

Supplementary Figure 1. Silhouette values across alternative clustering methods: the original MATLAB *K*-means approach, the analogous *K*-means approach repeated in python, Ward's hierarchical clustering, and nonlinear kernel *K*-means. Each method was repeated for $K = 2$ to $K = 10$, corresponding with the methods presented in the manuscript, and assessed for optimal separation of data. Higher silhouette values indicate better separation of data between clusters and small slopes between the K and $K+1$ solution indicate stability of the K solution. This metric indicates that the original MATLAB *K*-means optimally separates the data, three of the four metrics agree that $K = 6$ is a good solution, and kernel *K*-means performs poorly.

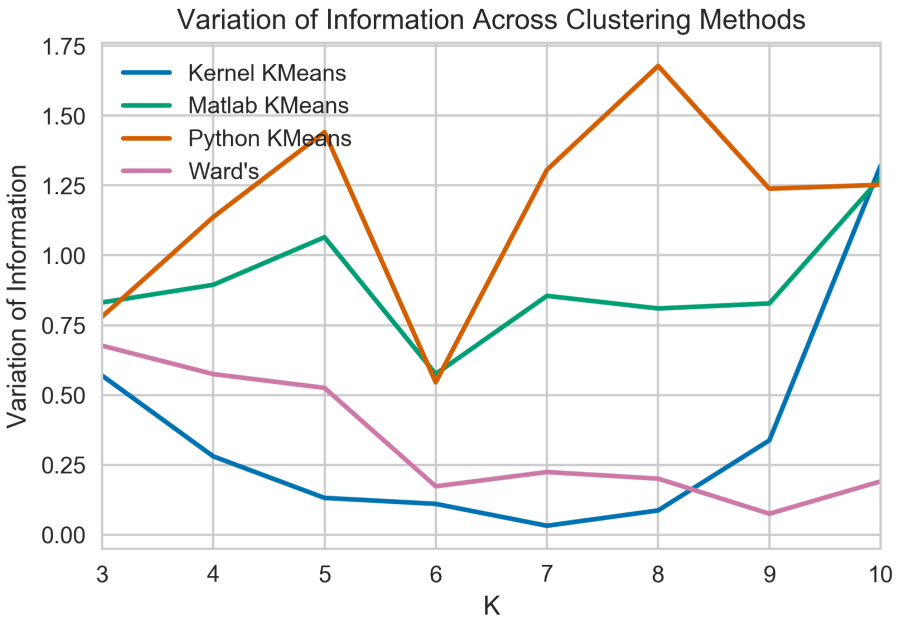

Supplementary Figure 2. Variation of information (VI) across clustering methods: the original MATLAB *K*-means approach, the analogous *K*-means approach repeated in python, Ward's hierarchical clustering, and nonlinear kernel *K*-means. Each method was repeated for $K = 2$ to $K = 10$, corresponding with the methods presented in the manuscript, and assessed for stability across clustering solutions. Local minima in the plot of VI against K indicate that K is a relatively stable solution, as seen at $K = 6$ for MATLAB *K*-means, python *K*-means, and Ward's hierarchical clustering. This corresponds with the choice of clustering solution presented in the manuscript, presenting an agreement across clustering methods that $K = 6$ is the most stable solution for this data.

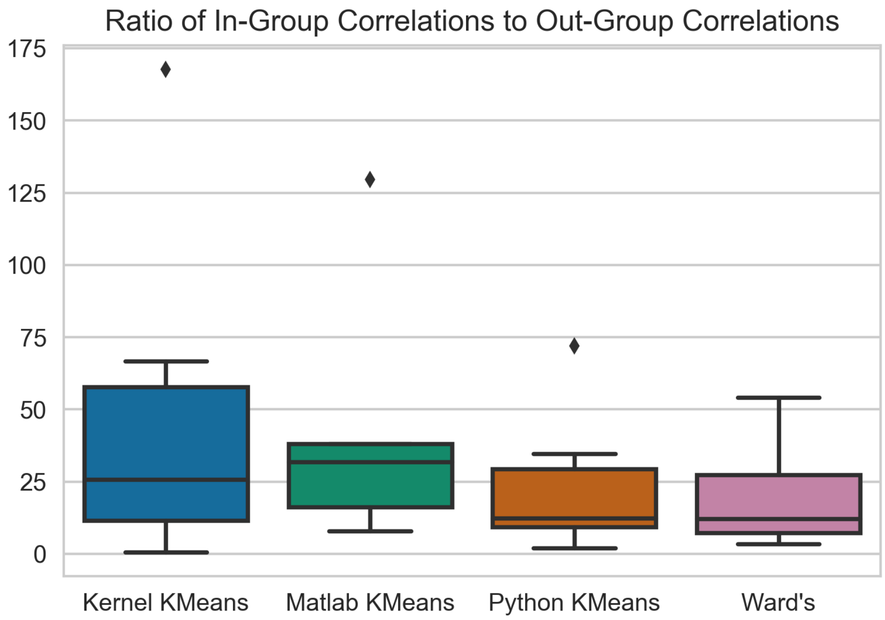

Supplementary Figure 3. The ratio of pairwise correlations between spatial patterns of activation for experiments within a cluster are than correlations between those experiments and experiments of other clusters, indicating within-group similarity as a ratio of in-group to out-group correlations. The highest median ratio is seen for the original MATLAB *K*-means approach, followed by kernel *K*-means, which performed poorly on the other metrics considered. A high ratio for MATLAB *K*-means indicates that the method described throughout the manuscript maximally grouped similar experiments and separated dissimilar experiments.
